# Factors related to non-compliance to HPV vaccination in Roraima—Brazil: a region with a high incidence of cervical cancer

**DOI:** 10.1186/s12913-016-1677-y

**Published:** 2016-08-22

**Authors:** Cibelle Carneiro Farias, Dkaion Vilela Jesus, Hendel Santana Moraes, Ingrid Ferreira Buttenbender, Isabella Seixas Martins, Mayara Gabrielle Souto, Paulo Henrique Brasil Hass Gonçalves Filho, Randielly Mendonça Costa, Sarah de Oliveira Silva, Thais Suelen Israel Ferreira, Valéria Vieira da Silva Coutinho, Helvia Rochelle Tavora Minotto, Allex Jardim Fonseca

**Affiliations:** 1 Medical student of Universidade Federal de Roraima, Boa Vista, Brazil; 2 Professor of Health Science of Universidade Federal de Roraima, Boa Vista, Brazil; 3 Department of Research on Health Science, Universidade Federal de Roraima, Boa Vista, Brazil; 4 Centro de Ciência da Saúde, UFRR. Secretaria do Curso de Medicina, Campus de Paricarana, s/n. Universidade Federal de Roraima, CEP: 69–307.000, Boa Visa, Roraima Brazil

**Keywords:** Papillomavirus, Vaccine, Vaccine coverage, Cervical cancer

## Abstract

**Background:**

To evaluate the HPV vaccination coverage in Boa Vista, Roraima (Brazil) and to identify personal and socioeconomic factors related to non-compliance to HPV vaccination.

**Methods:**

A school-based, cross-sectional study was conducted by distributing a self-administered questionnaire to the parents or guardians of pre-adolescent girls. The questionnaire addressed compliance to, knowledge about and perception of HPV and the HPV vaccine. Between July and November 2015, 13 private and public schools were visited based on a random cluster sampling method.

**Results:**

A total of 1337 questionnaires were distributed to all female students in the target age group, and 797 were completed and returned (the participation rate was 59.6 %). The vaccination coverage rate was 82.7 % and was higher among public school students than among private school students (84.1 % vs 56.3 %; *p =* 0.003). Most parents (60 %) incorrectly answered more than half of the questions related to HPV knowledge, and limited knowledge about HPV and the HPV vaccine correlated with lower compliance to vaccination (adjusted *OR* = 1.42; 95 % CI: 1.01 to 2.76). In the perception analysis, the belief that the HPV vaccine is important for the daughter was an important protective factor (adjusted *OR* = 0.62; 95 % CI: 0.23 to 0.93), and concern about adverse effects of the HPV vaccine was a risk factor for non-compliance (adjusted *OR* = 1.66; 95 % CI: 1.01 to 2.71). Family income, religion and education level of the parents or guardians did not correlate with compliance to vaccination.

**Conclusion:**

HPV vaccination coverage was high in Boa Vista, Brazil, but knowledge about the vaccine was deficient. This deficiency was associated with a distorted perception and was negatively associated with compliance to vaccination. Actions aimed at informing the public about the HPV vaccine, including its risks and benefits, are needed to attain higher vaccination coverage in Brazil.

**Electronic supplementary material:**

The online version of this article (doi:10.1186/s12913-016-1677-y) contains supplementary material, which is available to authorized users.

## Background

Cervical cancer (CC) is the second most common type of cancer among women in Brazil, following nonmelanoma skin cancer [1]. In particular, the state of Roraima (Brazilian Amazonian Region) stands out, as it presents one of the highest incidence rates of cervical cancer in the country. In 2010, a population-based study conducted in Roraima (Amazonian Region) showed a crude CC incidence rate of 46.2 per 100,000 women, surpassing the estimates for that state in that year by the National Cancer Institute (24/100,000 people) [2]. Moreover, the CC crude incidence rate of Roraima was comparable to that of low-income underdeveloped countries.

The main risk factor for the development of cervical cancer is infection with human papillomavirus (HPV), and the predominant route of transmission is sex. Approximately 12 HPV types are considered oncogenic, and types 16 and 18 are responsible for nearly 70 % of cervical cancer cases [3]. To establish a primary prevention strategy for cervical cancer, two cost-effective vaccines have been developed: a bivalent vaccine that protects against HPV types 16 and 18 and a quadrivalent vaccine that protects against types 6, 11, 16 and 18 [4]. In November 2013, the quadrivalent vaccine was incorporated into the immunization schedule of Brazil. The first stage of the free immunization program offered by the Unified Health System began in 2014 and included girls between 11 and 13 years old. This age group was chosen so that the antibodies were produced before exposure to the virus and so that the adolescents were protected against premalignant lesions of uterine cervical cancer.

Evidence shows that the HPV vaccine is safe and effective for the prevention of HPV infection [5]. However, as this vaccine targets a sexually transmitted disease and is offered to teens, some acceptance barriers exist, such as cultural factors, individual beliefs and knowledge and attitudes of parents and family members [6]. Another factor that may influence the choice to vaccinate is the insecurity of both girls and parents about adverse reactions to the vaccine. Stokley et al. [7] showed that the most common cause of non-vaccination among female adolescents in the United States of America was a lack of knowledge about the HPV vaccine. Another factor worth mentioning is that the target age of the HPV vaccine occurs after the period in which most children are vaccinated in Brazil, which is from 0 to 6 years old.

Attaining satisfactory vaccination coverage is the greatest challenge of HPV vaccination in Brazil. According to official data, the state of Roraima has achieved a participation rate of 85.7 % for the first dose and only 49.5 % for the second dose of the HPV vaccine [1]. The decreased participation rate for the second dose and the lack of studies on immunization have led to questions about which social groups have not adhered to HPV vaccination and why. The aim of this study was to evaluate vaccination coverage in Boa Vista, the capital of the state of Roraima, and to identify personal factors (knowledge and perception) and socioeconomic factors related to non-compliance to HPV vaccination.

## Methods

### Study design

This was a school-based cross-sectional study that used quantitative analysis to evaluate vaccination coverage and its determinants in Boa Vista between July and November 2015.

### Study population

Boa Vista is the capital of the state of Roraima and is located in the Brazilian Amazonian region. Boa Vista has 265,000 inhabitants, a concentrated population representing 65 % of all inhabitants of the state. In 2014, the city had 44 public schools and 12 private schools, attended by 9,974 and 2,300 female students within the target age range (6th to 9th grade), respectively. The study population was the parents or guardians of pre-adolescent girls (between 12 and 14 years of age in 2015) who were students of middle schools in the capital city Boa Vista.

### Sample and sampling

Every middle school in Boa Vista was registered and numbered. The sampling process consisted of random cluster sampling, assuming each school was a sample cluster. A sequence of random numbers was generated (http://www.random.org) to determine the order in which the schools were visited. The random sequence generation was weighted by the number of students (in the target age group) enrolled in each school.

Schools were visited according to the random sequence order until the target sample was achieved, which occurred at the thirteenth school. To calculate the sample size, the coverage of HPV vaccination was estimated to be 50 % based on official data. Considering a confidence interval of 95 % and an acceptable error of 5 %, a minimum sample size of 695 parents or guardians was obtained. This sample size had a power of 90 % to detect an adjusted odds ratio greater than 1.5 with a 95 % confidence interval (alpha error = 0.10, bilateral).

### Study procedures

The schools were visited after receiving approval by the board of each school. The researchers visited the classrooms of pre-adolescent girls who were within the target vaccination age range in 2014. After explaining the procedure, the students were asked to take a sealed envelope to their homes and to deliver it to their parents or guardians. The envelope contained a consent form and a self-administered questionnaire. The semistructured questionnaire addressed socioeconomic and demographic data in the first section (without personal identification). The questionnaire had been previously tested and validated with persons of different levels of education. The second section contained 6 questions that addressed the main outcome, the compliance to HPV vaccination and its circumstances. The third section consisted of 10 questions that evaluated the knowledge of the parents / guardians about HPV, cervical cancer and the HPV vaccine. The fourth and final section was composed of 8 assertive statements that assessed the parents’ perception of the HPV vaccine. The questionnaire, adapted from Ragin et al.[8], was previously tested. The research team returned to the school on the next day to collect the envelopes that contained questionnaires completed by the students’ parents / guardians. If the envelope return rate was less than 80 %, then the team returned to the school on subsequent days to collect the remaining questionnaires after providing additional invitation to participate. The period of data collection occurred from 1 July to 30 November, 2015. Parents of girls with contraindications to the vaccine or outside the target age range and parents who did not sign the consent form and/or did not complete the questionnaire were excluded.

## Data analysis method

### Variables

The primary outcome variable was non-compliance to vaccination, defined as the rate of girls between the ages of 11 and 13 in 2014 who either received at least 2 doses of the HPV vaccine (in 2014 or 2015) or received their first dose of the vaccine in early 2015. Personal and demographic data of the participating families were considered to be explanatory/descriptive variables.

### Statistical analysis

Descriptive analysis was performed and included a frequency distribution for categorical variables and means (with standard deviation) for continuous variables. The prevalence of the outcome variable and the 95 % confidence intervals (95 % CIs) were estimated based on a binomial distribution. To compare the sample means, we used Student’s *t* test for normally distributed variables when homogeneity of the sample variance was observed. Otherwise, the Mann–Whitney test was used for this purpose. We used the chi-square test to compare differences in the proportions of categorical variables. Odds ratios (ORs) and 95 % CIs were calculated for the univariate analysis, and adjusted odds ratios (aORs) were calculated for the multivariate logistic regression analysis. The criterion used to select explanatory variables for entry in the multivariate analysis model was the threshold value of *p <* 0.15 in the univariate analysis. The data were tabulated and analyzed using MedCalc® software (Oostende, Belgium).

### Ethical issues

This study was approved by the Research Ethics Committee of the Universidade Federal de Roraima. None of the invited school boards refused to participate. All participants signed the informed consent term.

## Results

Thirteen schools, 11 public and 2 private, were included. In these schools, all classrooms with girls within the target age range were visited and invited to participate. A total of 1,337 questionnaires were distributed, and 933 students returned the envelopes. Of these, 136 were discarded because they contained either a blank questionnaire or a blank consent form. The final sample consisted of 797 respondents (59.6 % participation rate).

The mean age of the students was 13.0 (±0.8) years. Only 12.6 % were studying in a private school (*n =* 101). The most common questionnaire respondent was the mother of the student (*n =* 601; 77.2 %), followed by the father (*n =* 88; 11.3 %). The mean age of the respondents was 39.1 years (±8.0), and most respondents had completed high school (*n =* 311; 41.0 %), followed by those who did not complete high school (*n =* 243; 32.2 %). It is noteworthy that only 24 respondents (3.1 %) declared themselves illiterate. The majority of respondents were Catholic (*n =* 277; 46.6 %), followed by Protestant (*n =* 262; 44.1 %) (Table 1). Approximately half of the respondents (*n =* 353) reported a monthly household income below I$400 (international dollars), which was the minimum wage at the time of the study.

**Table 1:** Sociodemographic characteristics of the sample

Characteristics	Mean (± SD)	n (%)
Student’s age (years old)	13,0 (±0,8)	
12		250 (32,7 %)
13		263 (34,4 %)
14		252 (32,9 %)
Survey Participant		
Parents		689 (88,5 %)
Grandparents		42 (5,4 %)
Uncle/Aunt		30 (3,9 %)
Guardian		17 (2,2 %)
Participant’s age	39,1 (±8,0)	
Up to 40 years old		485 (63,3 %)
More than 40 years old		281 (36,7 %)
Participant’s schooling		
illiterate		24 (3,1 %)
elementary school		180 (23,7 %)
high school		243 (32,2 %)
higher education		311 (41,0 %)
Private school		101 (12,6 %)
Public school		696 (87,4 %)
Participant religion		
Catholic		277 (46,6 %)
Protestant		262 (44,1 %)
Others		55 (9,3 %)

Among the 797 surveyed students, 758 (95.1 %) had received at least 1 dose of the HPV vaccine. Of these, 659 were considered to be adherent to vaccination (i.e., received 2 doses for those who began vaccination in 2014 or received 1 dose for those who began in 2015). Therefore, the rate of non-compliance to HPV vaccination was 17.3 % (95 % CI: 16.8 to 17.8 %), and this group was composed of 39 never-vaccinated girls and 99 girls in the waiting period to receive the second dose of the HPV vaccine. Considering only the cases with some type of non-compliance, 50 parents (36.2 %) reported that they intended to vaccinate their daughters to rectify the delay in vaccination during the year of the study. When asked about the cause of non-vaccination, the main reasons reported were concern about the side effects (14.8 %), forgetfulness (11.8 %), lack of time due to a busy life (7.4 %), and lack of knowledge about the vaccine (7.4 %). There was no significant difference in these reasons between public and private schools.

**Fig. 1:**
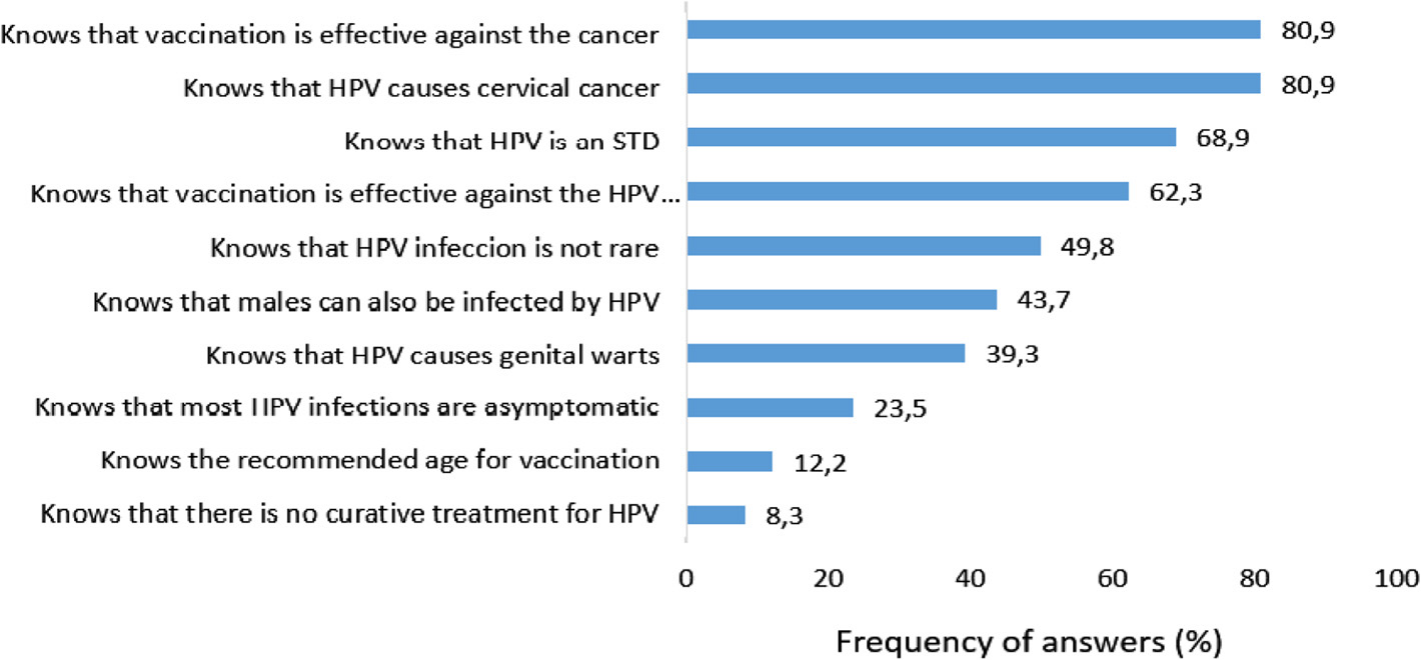
Description of knowledge of the respondents about HPV vaccination (10 issues)

On average, the respondents correctly answered 4.5 (±2.3) out of 10 questions about the HPV vaccine and cervical cancer. The questions that were most commonly answered correctly involved the causal relationship between HPV and cervical cancer (80.9 %) and the purpose of the vaccine, which was to prevent cervical cancer (80.9 %). Few respondents knew that HPV infection is usually asymptomatic (23.5 %), the recommended age for vaccination (12.2 %) and that there is no curative treatment for HPV infection (8.3 %) (Fig. 1).

Private school students had a significantly increased rate of non-compliance to HPV vaccination compared to public school students (43.7 % vs 15.9 %; *p =* 0.003; *OR* = 4.51, 95 % CI: 1.63 to 12.46). Other sociodemographic variables, such as religion, respondent education level and family income, did not correlate with the outcome variables studied. However, knowledge about the HPV vaccine and HPV correlated with compliance to HPV vaccination. Correctly answering less than 50 % of the knowledge-related questions increased the likelihood of non-compliance to vaccination by 60 % (20.2 % vs 13.6 %; *p =* 0.02, *OR* = 1.60; 95 % CI: 1.05 to 2.45). When each knowledge-related question was analyzed individually, the following items increased non-compliance: not knowing that HPV is common (21.5 % vs 13.1 %; *p =* 0.001), not knowing that the vaccine was effective in preventing HPV (20.6 % vs 14.0 %; *p =* 0.01), not knowing that the purpose of the HPV vaccine is to prevent cervical cancer (24.4 % vs 15.6 %; *p =* 0.01) and not knowing the recommended age for HPV vaccination (18.3 % vs 10.3 %; *p =* 0.04). Table 2 describes these results.

**Table 2:** Univariate analysis: correlation between demographic characteristics, parents’ knowledge about HPV vaccine and non-compliance to vaccination, Boa Vista, Brazil, 2015

Characteristics		Yes *n* (%)	Non-compliance to HPV vaccination		
			Prevalence	*p* value	Odds Ratio (CI 95 %)
Parent’s religion	Protestant	262 (44,1 %)	17,9 %	ns	1,03 (0,576–1,45)
	Non protestant	332 (55,9 %)	17,5 %		1
Parent’s schooling	Elementary school	203 (26,8 %)	16,7 %	ns	0,98 (0,71–1,73)
	High school / higher	554 (73,2 %)	16,9 %		1
Monthly household income	Up to I$ 400	353 (49,5 %)	16,1 %	ns	1,02 (0,61–1,41)
	More than I$ 400	360 (50,5 %)	18,6 %		1
Type of school	Private school	99 (12,5 %)	43,7 %	0,003	4,51 (1,63–12,46)
	Public school	698 (87,5 %)	15,9 %		1
Correct answers in 10 knowledge questions	Less than 50 %	287 (38,8 %)	20,2 %	0,02	1,60 (1,05–2,42)
	More than 50 %	453 (61,2 %)	13,6 %		1
Knowing that HPV infections is not rare	Wrong	400 (50,2 %)	21,5 %	0,001	1,81 (1,24–2,64)
	Right	397 (49,8 %)	13,1 %		1
Knowing the recommended age group for vaccination	Wrong	700 (87,8 %)	18,3 %	0,04	1,94 (1,02–3,86)
	Right	97 (12,2 %)	10,3 %		1
Knowing that the vaccine is effective in preventing HPV	Wrong	301 (37,7 %)	20,6 %	0,01	1,62 (1,08–2,41)
	Right	496 (62,3 %)	14,0 %		1
Knowing that the vaccine prevents cervical cancer	Wrong	152 (19,1 %)	24,4 %	0,01	1,73 (1,14–2,84)
	Right	645 (80,9 %)	15,6 %		1
Knowing that HPV is a STD	Wrong	248 (31,1 %)	17,7 %	ns	1,04 (0,73–1,66)
	Right	549 (68,9 %)	17,1 %		1
Knowing that men are affected by HPV	Wrong	449 (56,3 %)	18,0 %	ns	1,12 (0,71–1,60)
	Right	348 (43,7 %)	16,3 %		1
Knowing that HPV causes genital warts	Wrong	484 (60,7 %)	18,8 %	ns	1,41 (0,92–2,17)
	Right	313 (39,3 %)	15,0 %		1
Knowing that HPV causes CC	Wrong	152 (19,1 %)	19,7 %	ns	1,35 (0,85–2,16)
	Right	645 (80,9 %)	16,7 %		1
Knowing that the HPV infection is usually asymptomatic	Wrong	610 (76,5 %)	16,1 %	ns	0,71 (0,43–1,06)
	Right	187 (23,5 %)	21,4 %		1
Knowing that there is no curative treatment for HPV	Wrong	731 (91,7 %)	17,6 %	ns	1,53 (0,63–3,67)
	Right	66 (8,3 %)	13,6 %		1

*ns* not significant

Based on analysis of the correlation between the perception of the HPV vaccine and compliance to vaccination, the respondents who were concerned about the adverse effects of the vaccine showed a 60 % higher rate of non-vaccination (*OR* = 1.62, 95 % CI 1.04 to 2.51) than those who were not concerned about adverse events (19.3 % vs 12.8 %; *p =* 0.03). Those concerned with the quality of the vaccine freely offered by the Brazilian Unified Health System similarly showed an 80 % higher likelihood of non-vaccination (24.4 % vs 14.7 %; *p =* 0.003; *OR* = 1.78, 95 % CI: 1.15 to 2.99). The perception that the HPV vaccine is important for the health of the student was a protective factor. Parents who agreed with the importance of the vaccine had a significantly lower rate of non-compliance than those who disagreed (15.7 % vs 37.5 %, respectively; *p =* 0.004; *OR* = 0.31, 95 % CI: 0.13 to 0.73), reducing the likelihood of non-vaccination by approximately one-third. The other questions on perception did not correlate with the outcome variables. These analyses are presented in Table 3.

**Table 3:** Univariate analysis between parent’s perception on HPV vaccination and non-compliance to vaccination, Boa Vista, Brazil, 2015

Parent’s perception		Yes n (%)	Non-compliance to HPV vaccination		
			Prevalence	*p* value	Odds Ratio (95 % IC)
The quality of the vaccine offered by the government is worrying	Agrees	549 (77,4 %)	24,4 %	0,003	1,78 (1,15–2,99)
	Disagrees	160 (22,6 %)	14,7 %		1
The vaccine is important for my daughter’s health	Agrees	691 (96,6 %)	15,7 %	0,004	0,31 (0,13–0,73)
	Disagrees	24 (3,4 %)	37,5 %		1
The adverse effects of the vaccine are worrying	Agrees	461 (64,9 %)	19,3 %	0,03	1,62 (1,04–2,51)
	Disagrees	249 (35,0 %)	12,8 %		1
The vaccine can induce early sexual initiation in girls	Agrees	137 (19,4 %)	20,4 %	ns	1,40 (0,86–2,29)
	Disagrees	571 (80,6 %)	16,1 %		1
The age range recommended for the vaccine is too early	Agrees	226 (31,9 %)	18,5 %	ns	1,17 (0,71–1,78)
	Disagrees	481 (60,1 %)	14,9 %		1
The vaccine can induce unsafe sex	Agrees	184 (25,9 %)	17,4 %	ns	1,05 (0,67–1,83)
	Disagrees	525 (74,1 %)	16,5 %		1
Parents have to talk about sex with their daughters before vaccination	Agrees	664 (93,2 %)	16,1 %	ns	0,72 (0,33–1,55)
	Disagrees	43 (6,8 %)	20,1 %		1
The decision to vaccinate belongs to the students, not to the parents	Agrees	187 (26,5 %)	15,5 %	ns	0,91 (0,57–1,49)
	Disagrees	518 (73,5 %)	16,8 %		1

*ns* not significant

The variables that correlated with non-compliance to the HPV vaccine based on the univariate analysis were re-evaluated in the multivariate analysis. In this analysis, attendance of a private school was the main risk factor (adjusted *OR* = 2.44; 95 % CI: 1.33 to 4.31), followed by concern about the adverse effects of the vaccine (adjusted *OR* = 1.66; 95 % CI: 1.01 to 2.71) and having little knowledge about HPV and the HPV vaccine (adjusted *OR* = 1.42; 95 % CI: 1.01 to 2.76). In contrast, the knowledge that HPV infection is common remained as a protective factor (adjusted *OR* = 0.52; 95 % CI: 0.33 to 0.81) and the belief that the vaccine is important for the health of the student reduced the chance of non-compliance by approximately 40 % (adjusted *OR* = 0.62; 95 % CI: 0.23 to 0.93). The other selected variables were not confirmed to be factors related to vaccination (Table 4). The spreadsheet containing raw data is available in Additional file 1.

**Table 4:** Multivariate analysis for non-compliance to vaccination against HPV, Boa Vista – Brazil, 2015

Explanatory variables	Adjusted odds ratio (95% IC) for non-compliance to vaccination	*P* value
Private school student	2,44 (1,33–4,31)	0,002
Scoring less than 50% of the questions about HPV and the vaccine	1,42 (1,01–2,76)	0,045
Knowing that the HPV infection isn’t rare	0,52 (0,33–0,81)	0,004
Knowing that the vaccine is effective in preventing HPV infection	0,82 (0,51–1,31)	ns
Knowing that the vaccine is effective in preventing cervical cancer	0,84 (0,44–1,60)	ns
Believing that the adverse effects of the vaccine are worrying	1,66 (1,01–2,71)	0,04
Believing that the quallity of the vaccine provided by the government is worrying	1,51 (0,85–2,39)	ns
Believing that the vaccine is important for their daughter’s health	0,62 (0,23–0,93)	0,03

*ns* not significant.

## Discussion

This was the first study that evaluated the non-compliance to HPV vaccination in Brazil. The rate of non-compliance to vaccination (2 doses) in our sample was relatively high, at 17,3 %. A similar study conducted in Japan (Fukuoka) in 2015 also presented a self-administered questionnaire to parents and guardians of 2,097 students and reported a similar rate of non-compliance to free HPV vaccination (21,6 %) [9]. Holman et al. [10] conducted a systematic review of HPV vaccine compliance. The authors described a higher non-compliance rate (30 %), which was even higher within segments of the population without health insurance. In Brazil, there are no similar scientific studies for comparison. However, a study conducted in the city of Barretos (São Paulo, Brazil) evaluated the acceptance of the vaccine among parents of pre-adolescent girls in 2012 (before the availability of the free HPV vaccine in Brazil) and reported interesting results [11]. Similar to our study, Fregnani et al. conducted a school-based study but included an intervention: they invited parents and guardians of students to a meeting, where the attendees were informed about the vaccine. The authors reported that approximately 92 % of the parents authorized vaccination after the meeting. These studies reported a 97.2 % compliance rate of HPV vaccination, which was higher than the rate observed in our study. This evidence highlights the need to integrate health services with the school system to achieve higher coverage.

Multiple factors, such as issues related to the availability and distribution system for vaccines, cultural factors, family influences and socioeconomic factors, may influence compliance to vaccination [12]. However, the knowledge and perception of parents regarding preventive immunization strategies against HPV played a key role in compliance to vaccination. In our sample, most of the respondents knew the answers to less than half of the questions about the HPV vaccine and HPV. In 2011, a similar study conducted in the Basic Health Units of Campinas (São Paulo, Brazil) interviewed a total of 538 parents of girls [13]. Among these, only 40 % had heard about HPV, 29 % reported having adequate information about HPV, and less than 10 % knew about the HPV vaccine. The authors also reported that the parents who give more correct answers to the HPV knowledge-related questions showed greater intention to obtain the vaccination.

This relationship between knowledge about and compliance to vaccination was also demonstrated in our study. Parents and guardians with higher performance on the knowledge questions had an increased likelihood of vaccination compliance. One aim of this study was to identify which aspects of knowledge related to HPV had a significant influence on the decision to vaccinate. In our sample, the facts with the greatest influence were knowing that HPV infection is not rare, that the HPV vaccine is effective and that its purpose is to prevent cervical cancer. In addition, the perception of parents that the vaccine is important for the health of their daughter was a protective factor for vaccination. Corroborating these results, Osis et al. [13] reported that there was a high acceptance of fathers and mothers to vaccinate their daughters after receiving detailed information about the HPV vaccine among patients of the Unified Health System of Campinas, São Paulo. In a study by Fregnani et al. [11] (Barretos, Brazil), parents stated that a significant reason that discouraged them from obtaining the HPV vaccine was misinformation. There is thus a need for actions that promote public awareness and information about immunization. It is also important to rethink the quality and the vehicle of this information.

It was evident that attitudinal questions also substantially influenced this process. A portion of the respondents did not vaccinate their daughters due to forgetfulness or lack of time, which were the second and third leading causes of vaccine evasion in our study, respectively. Regarding the causes of forgetfulness, Ramos et al. [14] reported a sense of psychological tranquility caused by a long period without vaccination, which mainly occurred for vaccines that required more than one dose. In addition, the fact that the vaccine was recommended for an age range outside the usual age range of vaccination in Brazil represented another barrier to HPV vaccination. In Japan, it has also been reported that HPV vaccination was primarily promoted by passively targeting mothers rather than targeting girls by positively promoting the prevention of cervical cancer [9].

Stokley et al. [7] analyzed more than 25,000 adverse event reports of HPV vaccination in the United States. Although they reported that 92.4 % of these adverse events were limited to mild, such as local reactions, nausea, headache and syncope in response to the vaccine, and although the vaccine was classified as safe, there has been a growing anti-vaccine movement throughout the world [15]. Several studies have been published in recent years highlighting the harmful effects of the anti-vaccine movement, which has adversely affected individuals and populations [16, 17]. Rather than offer a legitimate explanation of the actual risks and benefits of vaccines, some authors and personalities have preferred to classify them as “weapons of mass destruction” [18]. In fact, studies in several countries, such as Vietnam [19], Japan [20], and the United States [15], have shown that concern about adverse effects is one of the most important factors influencing HPV vaccination non-compliance. Such concerns were a major cause of non-compliance in our study based on the greater than 60 % increase in the likelihood of non-compliance among those with these concerns. Additionally, the perception that the HPV vaccine provided freely by the Brazilian Unified Health System was of poor quality was also associated with significant risk of non-compliance. The study by Fregnani et al. [11] showed that although no serious adverse effects of the HPV vaccine have been reported, the fear of serious adverse effects was one of the main reasons that influenced subjects to avoid participating in the vaccination offered by the study.

Compliance to vaccination was more frequent among public school students than among private school students in our sample. There may be socioeconomic differences between the parents of students from these two types of schools, and these factors may influence the acceptance of the vaccine. One study by Osis et al. [13] in Campinas reported that people with lower education levels (less than 9 years of education) had less knowledge about HPV than people with nine or more years of education. Furthermore, greater knowledge about HPV was observed among those with higher social status and higher income. In Japan, the highest household income also correlated with increased acceptance of vaccination by mothers [9]. However, in our study, income, education and religion of the parents and guardians did not correlate with HPV vaccination compliance.

This study has limitations. First, the cross-sectional design did not permit the use of temporality as a criterion of causality because risk factors and outcome variables were measured synchronously and because the bias of reverse-causality could not be eliminated. Second, the difference in vaccine coverage between private and public schools is difficult to explain, especially because there was no evident difference in questionnaire responses regarding the causes of non-compliance between these groups. Finally, the use of questionnaires for data collection related to risk factors may be inaccurate due to concealment of the truth and answer masking. Moreover, there is the possibility of selection bias inherent in the return of the questionnaires, which may have encouraged more of those who were vaccinated to respond. However, the achieved sample target and the random selection of schools strengthened the credibility of the results.

## Conclusion

We conclude that the rate of compliance to HPV vaccination was high in Boa Vista, Brazil. Knowledge about the HPV vaccine and cervical cancer was deficient among parents and guardians of pre-teen girls, and this deficiency negatively influenced compliance to vaccination. Attitudinal deficits and concerns about the adverse effects of the HPV vaccine were also substantial barriers to vaccination. HPV vaccination was less frequent in private schools than in public schools, and further analysis is needed to confirm and understand this relationship. Our data suggest that programs aimed at informing the poorest and richest segments of the population about HPV vaccination, including its risks and benefits, are needed to fill knowledge gaps, to eliminate distortions of perception and to attain high HPV vaccination coverage to control cervical cancer.

## Additional file

Additional file 1: Spreadsheet containing raw data of this study. (XLS 1492 kb)

## Abbreviations

aOR: Adjusted odds ratio
CC: Cervical cancer
HPV: Human papillomavirus
OR: Odds ratio

## References

[1] Instituto Nacional de Câncer. Ministério da Saúde. Brasil Estimativa 2016: Incidência de câncer no Brasil. In*.* Edited by Saúde. Md, vol. 2016. Rio de Janeiro.: Editora INCA; 2016.

[2] Da Fonseca AJ, Ferreira LP, Dalla-Benetta AC, Roldan CN, Ferreira ML (2010). Epidemiology and economic impact of cervical cancer in Roraima, a Northern state of Brazil: the public health system perspective. Rev Bras Ginecol Obstet.

[3] Chen Z, De Freitas LB, Burk RD (2015). Evolution and classification of oncogenic human papillomavirus types and variants associated with cervical cancer. Methods Mol Biol.

[4] Paavonen J, Jenkins D, Bosch FX, Naud P, Salmeron J, Wheeler CM, Chow SN, Apter DL, Kitchener HC, Castellsague X (2007). Efficacy of a prophylactic adjuvanted bivalent L1 virus-like-particle vaccine against infection with human papillomavirus types 16 and 18 in young women: an interim analysis of a phase III double-blind, randomised controlled trial. Lancet.

[5] Gee J, Weinbaum C, Sukumaran L, Markowitz LE. Quadrivalent HPV vaccine safety review and US safety monitoring plans for nine-valent HPV vaccine. Human vaccines immunotherapeutics. 2016. 10.1080/21645515.2016.1168952 PMC4964727 27029786

[6] Ferrer HB, Trotter C, Hickman M, Audrey S (2014). Barriers and facilitators to HPV vaccination of young women in high-income countries: a qualitative systematic review and evidence synthesis. BMC Public Health.

[7] Stokley S, Jeyarajah J, Yankey D, Cano M, Gee J, Roark J, Curtis RC, Markowitz L (2014). Human papillomavirus vaccination coverage among adolescents, 2007–2013, and postlicensure vaccine safety monitoring, 2006-2014--United States. MMWR Morb Mortal Wkly Rep.

[8] Ragin CC, Edwards RP, Jones J, Thurman NE, Hagan KL, Jones EA, Moss CM, Smith AC, Akers A, Gollin SM (2009). Knowledge about human papillomavirus and the HPV vaccine--a survey of the general population. Infectious agents and cancer.

[9] Hamada Y, Nagamatsu M, Sato T (2015). Factors Influencing Maternal Acceptance of Human Papillomavirus Vaccination for Their School-Aged Daughters in Fukuoka Prefecture, Japan. Br J Med Med Res.

[10] Holman DM, Benard V, Roland KB, Watson M, Liddon N, Stokley S (2014). Barriers to human papillomavirus vaccination among US adolescents: a systematic review of the literature. JAMA Pediatr.

[11] Fregnani JH, Carvalho AL, Eluf-Neto J, Ribeiro Kde C, Kuil Lde M, Da Silva TA, Rodrigues SL, Mauad EC, Longatto-Filho A, Villa LL (2013). A school-based human papillomavirus vaccination program in barretos, Brazil: final results of a demonstrative study. PLoS One.

[12] Juliano Y, Compri PC, Almeida LR, Freire PV, Moreira FT, Vieira FHS, Rossi S, Figueira K (2008). Segunda etapa da Campanha Nacional de Multivacinação do município de São Paulo, 2005: perfil de cobertura de diferentes Unidades Básicas de Saúde. Revista Paulista de Pediatria.

[13] Osis MJD, Duarte GA, Sousa MH (2014). Conhecimento e atitude de usuários do SUS sobre o HPV e as vacinas disponíveis no Brasil. Rev Saude Publica.

[14] Ramos CF, Paixão JGM, Donza FCS, Silva AMP, Caçador DF, Dias VDV, Sodré ÉFLdM (2010). Cumprimento do calendário de vacinação de crianças em uma unidade de saúde da família. Revista Pan-Amazônica de Saúde.

[15] White MD (2014). Pros, cons, and ethics of HPV vaccine in teens-Why such controversy?. Transl Androl Urol.

[16] Gangarosa EJ, Galazka AM, Wolfe CR, Phillips LM, Gangarosa RE, Miller E, Chen RT (1998). Impact of anti-vaccine movements on pertussis control: the untold story. Lancet.

[17] Jansen VA, Stollenwerk N, Jensen HJ, Ramsay ME, Edmunds WJ, Rhodes CJ (2003). Measles outbreaks in a population with declining vaccine uptake. Science.

[18] Poland GA, Jacobson RM, Ovsyannikova IG (2009). Trends affecting the future of vaccine development and delivery: the role of demographics, regulatory science, the anti-vaccine movement, and vaccinomics. Vaccine.

[19] Paul P, LaMontagne DS, Le NT (2012). Knowledge of cervical cancer and HPV vaccine post-vaccination among mothers and daughters in Vietnam. Asian Pac J Cancer Prev.

[20] Hanley SJ, Yoshioka E, Ito Y, Konno R, Hayashi Y, Kishi R, Sakuragi N (2012). Acceptance of and attitudes towards human papillomavirus vaccination in Japanese mothers of adolescent girls. Vaccine.

